# Predicting upper limb motor dysfunction after ischemic stroke: a functional near-infrared spectroscopy-based nomogram model

**DOI:** 10.3389/fneur.2025.1524851

**Published:** 2025-05-27

**Authors:** Menghui Liu, Chunxiao Wan, Chunyan Wang, Xinyi Li

**Affiliations:** Department of Rehabilitation Medicine, Tianjin Medical University General Hospital, Tianjin, China

**Keywords:** ischemic stroke, upper limb motor function, functional near-infrared spectroscopy, predictive model, nomogram

## Abstract

**Background:**

This study aimed to identify independent risk factors associated with upper limb motor functional recovery in ischemic stroke patients 3 months post-stroke and to construct a predictive model based on functional near-infrared spectroscopy (fNIRS) data.

**Methods:**

The study included 114 patients with ischemic stroke, divided into a training group (*n* = 80) and a validation group (*n* = 34). Variables related to the FMA-UE score 3 months later were selected from fNIRS data using LASSO regression, and independent risk factors were determined through logistic regression analysis. A nomogram was constructed based on these factors to predict the probability of upper limb motor dysfunction scores after stroke, and the model’s discriminative ability was assessed using the area under the ROC curve (AUC), as well as the clinical net benefit was evaluated using decision curve analysis (DCA).

**Results:**

The LASSO regression ultimately selected seven variables for the assessment of motor dysfunction post-stroke, of which five were identified as independent risk factors. The five independent fNIRS risk factors associated with upper limb motor functional recovery are A_A_dxy_DLPFC_to_Temporal: The number of brain functional connectivity edges from the affected side dorsolateral prefrontal cortex (DLPFC) to the affected side temporal lobe under deoxygenated hemoglobin monitoring level, A_UA_oxy_DLPFC_to_PSMC: The number of brain functional connectivity edges from the affected side DLPFC to the unaffected side primary somatosensory motor cortex (PSMC) under oxyhemoglobin monitoring level, A_UA_total_Temporal_to_DLPFC: The number of brain functional connectivity edges from the affected side temporal lobe to the unaffected side DLPFC under total hemoglobin monitoring level, UA_UA_dxy_Temporal_to_Frontopolar: The number of brain functional connectivity edges from the unaffected side temporal lobe to the unaffected side frontopolar cortex under deoxygenated hemoglobin monitoring level, and UA_UA_total_PSMC_to_PMC: The number of brain functional connectivity edges from the unaffected side PSMC to the unaffected side premotor cortex (PMC) under total hemoglobin monitoring level. The AUC of the ROC curve for the nomogram was 0.971 in the training dataset and 0.804 in the testing dataset, demonstrating good discriminative ability. DCA results indicated that the model showed good clinical net benefit in both the validation and development cohorts.

**Conclusion:**

This pilot study successfully constructed a predictive model based on fNIRS data to forecast the risk factors for upper limb motor functional recovery 3 months after ischemic stroke, providing a valuable tool for clinical decision-making and treatment planning.

## 1 Introduction

Ischemic stroke is one of the leading causes of adult disability worldwide (1–3), severely impacting patients’ quality of life (4–6). The recovery of upper limb motor function is crucial for patients’ activities of daily living. Functional near-infrared spectroscopy (fNIRS), as a non-invasive neuroimaging technique, can monitor brain activity in real-time, offering a new perspective for assessing the recovery of upper limb motor function after stroke (7–9). Although studies have explored the application of fNIRS in stroke rehabilitation, most have focused on short-term effects (10), and there is a lack of predictive models for long-term outcomes. Therefore, this study aims to identify independent risk factors associated with the recovery of upper limb motor function in ischemic stroke patients 3 months post-stroke through fNIRS data and to construct a predictive model, in hopes of providing a more precise assessment tool for clinical practice and guiding the formulation of rehabilitation treatment plans.

## 2 Materials and methods

### 2.1 Patients

This study was approved by the Ethics Committee of the General Hospital of Tianjin Medical University (Ethics number: IRB2022-YX-054-01) and strictly adhered to the principles of the Declaration of Helsinki. As this was a retrospective study, the requirement for obtaining informed consent from participants was waived. Prior to analysis, all patient medical information was anonymized to protect privacy. The study included 353 ischemic stroke patients treated in the Rehabilitation Department of the General Hospital of Tianjin Medical University from October 2023 to July 2024.

The inclusion criteria for the study were: (1) A confirmed diagnosis of ischemic stroke; (2) Informed consent provided by the patient or their legal representative; (3) Age 18 years or older. The exclusion criteria included: (1) Patients unable to complete follow-up after 3 months; (2) Patients with severe aphasia, cognitive impairment, or consciousness disturbance, who could not cooperate with the examination; and (3) Patients with incomplete or substandard functional near-infrared spectroscopy (fNIRS) data. Based on these criteria, a total of 114 patients were included in the study.

We categorized patients with an improvement of 9 points or more in the FMA-UE score 3 months after discharge into the Symptom Improvement Group (SIG), and those with less than a 9-point improvement into the Symptom Non-Improvement Group (SNIG) (11–13). There were 80 cases in SIG and 34 in SNIG. Patients were randomly assigned to the training group (80 patients, including 55 SIG and 25 SNIG) and the validation group (34 patients, including 25 SIG and 9 SNIG) in a 7:3 ratio.

### 2.2 Data collection

Data collection for this study was divided into three main parts: Firstly, basic patient information was extracted from the electronic medical record system, including age, gender, blood pressure levels at admission, time interval after stroke onset, body mass index (BMI), history of diabetes, history of hypertension, hemisphere of brain lesion, history of coronary heart disease, and FMA-UE score and modified Barthel Index at admission. Secondly, FMA-UE scores and modified Barthel Index were assessed through telephone or on-site follow-up 3 months after patient discharge. Lastly, functional connectivity data of the brain in a resting state for 5 min were collected using functional near-infrared spectroscopy (fNIRS) technology (model BS-20000s, 106 leads, produced by Wuhan Yiruid Company), dividing the brain into 12 regions of interest (including healthy and affected DLPFC, Temporal, Frontopolar, PSMC, SMA, and PMC), and counting the number of edges between all possible functional connections (a total of 279 variables) among these 12 regions.

The main fNIRS data variables in this study are oxy(oxyhemoglobin), dxy(deoxygenated hemoglobin), and total (total hemoglobin). Their calculation is based on the principles of fNIRS technology. First, the fNIRS device detects near-infrared light signals in the cerebral cortex (14, 15). Then, through a series of data processing steps, the raw near-infrared light intensity data is converted into optical density signals. Subsequently, motion artifact detection and correction are carried out using the HOMER2 toolbox (version 2.8) in MATLAB R2014b. Specifically, the built-in function of HOMER2 is used for motion artifact detection by channel (parameter settings are tMotion = 0.5 s; tMAsk = 3.0; STDEVthresh = 20.0; AMPthresh = 5.0), and the spline interpolation method (hmrMotionCorrectSpline) is used to detect and correct motion artifacts. Afterwards, a band-pass filter (0.01–0.1 Hz) is used to remove most systemic hemodynamic components, such as those originating from the cardiac cycle (about 1 Hz) and respiration (about 0.2–0.3 Hz). Finally, the filtered optical density data is converted to oxy, dxy, and total by applying the modified Beer–Lambert law. These variables are used to analyze the changes in resting-state functional connectivity in different brain regions during the iTBS intervention process.

### 2.3 Statistical analysis

Statistical analysis was conducted using SPSS software (version 27.0 by IBM Corporation) and R language (version 4.2.1). Initially, the Kolmogorov–Smirnov test was employed to assess the normality of continuous variables, which indicated that none of the variables followed a normal distribution. Consequently, medians and interquartile ranges were utilized to describe these variables, and the Mann–Whitney U test was applied to compare differences between groups. For changes in scores before and after treatment, the Wilcoxon signed-rank test was used for paired samples. Categorical variables were presented as frequencies (percentages) and analyzed using the chi-square test to determine statistical significance at a *p*-value less than 0.05 (two-tailed).

In the training set, we first employed LASSO regression to identify fNIRS data variables associated with FMA-UE scores 3 months later. Cross-validation was used to determine the optimal penalty parameter λ in LASSO regression to balance the model’s bias and variance. Specifically, we adopted 10 – fold cross – validation. The dataset was divided into 10 parts, and we took turns using 9 of them as the training set and 1 as the validation set. For each value of λ, we calculated the mean squared error (MSE) of the model on the validation set. Then, we averaged the MSEs obtained from the 10 – fold cross – validation to get the average mean squared error corresponding to each λ value. The λ value that minimized the average mean squared error was selected as the optimal λ. This approach was used to balance the model’s bias and variance and ensure that the model has good predictive ability on new data. Subsequently, logistic regression was conducted with upper limb motor dysfunction scores post-stroke as the dependent variable and variables selected by LASSO regression as independent variables to identify independent predictive factors. To ensure model stability, we assessed multicollinearity among variables using variance inflation factor (VIF) analysis. Based on the independent predictive factors determined by logistic regression analysis, a nomogram was constructed to predict the probability of upper limb motor dysfunction scores post-stroke. This nomogram will provide an intuitive tool to assess the risk of functional impairment based on specific patient characteristics. We evaluated the calibration performance of the model through calibration curve analysis, ensuring that the model’s predicted probabilities matched the actual observed probabilities. Additionally, decision curve analysis (DCA) was performed to assess the clinical net benefit of the model, involving a trade-off between potential harms and benefits. All model building was completed in R language (version 4.2.1). We used the “glmnet” package for LASSO regression to identify significant predictive variables; the “car” package to test VIF and assess multicollinearity; the “rms” package to construct the nomogram for predicting upper limb motor dysfunction scores post-stroke; the “pROC” package to obtain the C-index for both development and validation cohorts, evaluating the model’s discriminative ability; the “PredictABEL” package to assess improvements in predictive performance across different models, including IDI and NRI; and the “rmda” package for DCA to evaluate the clinical value of the developed nomogram.

## 3 Results

### 3.1 Baseline characteristics

**Figure 1:**
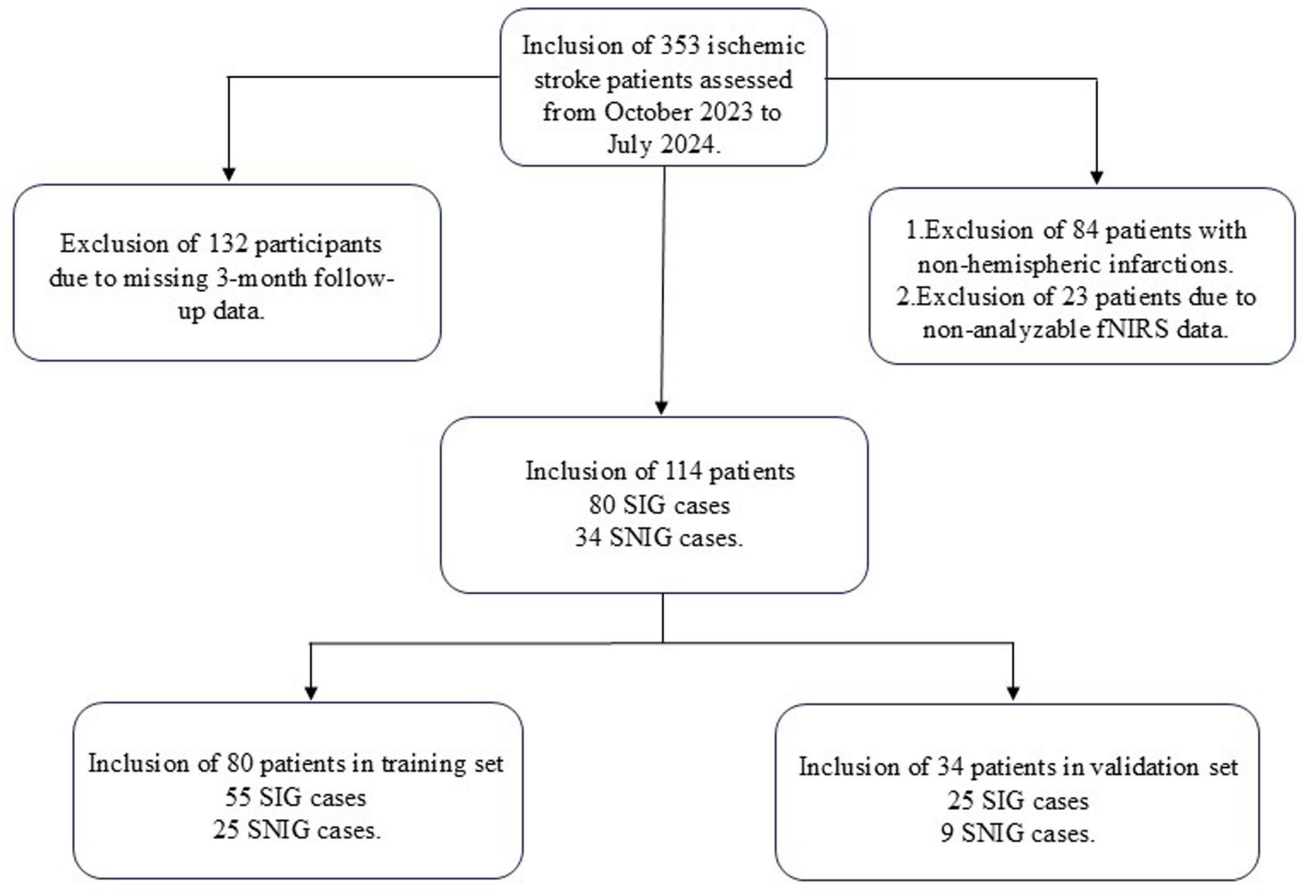
Flow diagram of the study.

From October 2023 to July 2024, a total of 353 patients with ischemic stroke were screened. After applying the inclusion and exclusion criteria, 114 patients met the criteria and were included in our study. Their basic information can be found in Table 1. These patients were then randomly assigned to the training group (80 patients) and the validation group (34 patients). The above process is shown in detail in Figure 1. The baseline characteristics of patients in both groups, including gender, age, disease duration, medical history, FMA-UE scores, and MBI scores before treatment, were not statistically significantly different (*p*-values >0.05), confirming that the randomization process for assigning patients to different groups was appropriate and reasonable. Specific baseline characteristic comparison data can be viewed in Table 2.

**Table 1:** Baseline characteristics of subjects.

Variables	Total (*N* = 114)	SIG (*N* = 80)	SNIG (*N* = 34)	χ^2^/Z	*P*
Sex, n (%)				0.015^a^	0.903
Male	56 (49.12)	39 (48.75)	17 (50)		
Female	58 (50.88)	41 (51.25)	17 (50)		
High blood pressure, n (%)				0.265^a^	0.607
Yes	14 (12.28)	9 (11.25)	5 (14.71)		
No	100 (87.72)	71 (88.75)	29 (85.29)		
Heart disease, n (%)				0.286^a^	0.593
Yes	68 (59.65)	49 (61.25)	19 (55.88)		
No	46 (40.35)	31 (38.75)	15 (44.12)		
Diabetes, n (%)				0.000^a^	0.985
Yes	20 (17.54)	14 (17.50)	6 (17.65)		
No	94 (82.46)	66 (82.50)	28 (82.35)		
History of stroke, n (%)				0.977^a^	0.323
Yes	87 (76.32)	59 (73.75)	28 (82.35)		
No	27 (23.68)	21 (26.25)	6 (17.65)		
Diseased hemisphere, n (%)				0.013^a^	0.910
Left	83 (72.81)	58 (72.50)	25 (73.53)		
Right	31 (27.19)	22 (27.50)	9 (26.47)		
Age (years)	66.50 (62, 72)	66.5 (62, 71.75)	67.5 (61.5, 72.5)	−0.468^b^	0.640
BMI (kg/m^2^)	24.48 (23.38, 25.39)	24.36 (23.53, 35.28)	25.16 (23.23, 26.42)	01.257^b^	0.209
CD (days)	53.5 (26, 90.25)	55 (30, 85.5)	48.5 (20.75, 90.5)	−0.325^b^	0.745
SP (mmHg)	124.5 (117.75, 132.25)	123 (117.25, 131)	126.5 (117, 134)	−0.639^b^	0.523
DP (mmHg)	78 (68.75, 87.25)	78.5 (68.25, 88)	77.5 (71.75, 85.50)	−0.412^b^	0.680
Before-FMA-UE	19.5 (8, 35)	18.5 (7, 30.75)	28 (11.5, 48.25)	−1.843^b^	0.065
After-FMA-UE	46 (27.75, 57)	48 (34.25, 58.75)	34.5 (15, 53)	−3.507^b^	<0.001
Before-MBI	20 (10, 49.25)	20 (10, 48.75)	20 (5, 46.25)	−0.476^b^	0.634
After-MBI	65 (45, 85)	70 (45, 85)	62.5 (38.75, 81.25)	−1.111^b^	0.267

CD, courses of disease; SP, systolic pressure; DP, diastolic pressure. FMA-UE, Fugl-Meyer Assessment Upper Extremity. MBI, Modified Barthel Index. ^a^ Using the χ^2^ test; ^b^ Using the Mann–Whitney U test.

**Table 2:** The baseline characteristics of the patients in the training and validation cohort.

Variables	Total (*N* = 114)	Training (*N* = 80)	Validation (*N* = 34)	χ^2^/Z	*P*
Diagnosis, n (%)				0.260^a^	0.610
SIG	80 (70.18)	55 (68.75)	25 (73.53)		
SNIG	34 (29.82)	25 (31.25)	9 (26.47)		
Sex, n (%)				0.886^a^	0.347
Male	56 (49.12)	37 (46.25)	15 (44.12)		
Female	58 (50.88)	43 (53.75)	19 (55.88)		
High blood pressure, n (%)				0.265^a^	0.607
Yes	14 (12.28)	9 (11.25)	5 (14.71)		
No	100 (87.72)	71 (88.75)	29 (85.29)		
Heart disease, n (%)				0.286^a^	0.593
Yes	68 (59.65)	44 (55)	19 (55.88)		
No	46 (40.35)	36 (45)	15 (44.12)		
Diabetes, n (%)				1.200^a^	0.273
Yes	20 (17.54)	12 (15)	8 (22.53)		
No	94 (82.46)	68 (85)	26 (76.47)		
History of stroke, n (%)				3.808^a^	0.051
Yes	87 (76.32)	57 (71.25)	30 (88.24)		
No	27 (23.68)	23 (23.75)	4 (11.76)		
Diseased hemisphere, n (%)				0.652^a^	0.420
Left	83 (72.81)	60 (75)	23 (67.65)		
Right	31 (27.19)	20 (25)	11 (32.25)		
Age (years)	66.50 (62, 72)	66 (62, 71)	68 (59.5, 74.25)	−0.800^b^	0.424
BMI (kg/m^2^)	24.48 (23.38, 25.39)	24.51 (23.43, 25.37)	24.37 (23.35, 25.81)	−0.266^b^	0.790
CD (days)	53.5 (26, 90.25)	52.5 (27.75, 90.75)	56.5 (24.75, 90.75)	−0.065^b^	0.948
SP (mmHg)	124.5 (117.75, 132.25)	125 (118.25, 132.5)	123.5 (114.75, 132.25)	−0.694^b^	0.487
DP (mmHg)	78 (68.75, 87.25)	80 (69, 87.75)	74 (63, 86.75)	−1.273^b^	0.203
Before-FMA-UE	19.5 (8, 35)	22 (10, 37.75)	14.5 (6, 29.25)	−1.562^b^	0.118
After-FMA-UE	46 (27.75, 57)	45.5 (26.25, 57)	48 (32, 57)	−0.313^b^	0.754
Before-MBI	20 (10, 49.25)	20 (6.25, 48.75)	25 (13.75, 38.75)	−0.261^b^	0.794
After-MBI	65 (45, 85)	70 (45, 85)	62.5 (45, 81.25)	−0.106^b^	0.916

CD, courses of disease; SP, systolic pressure; DP, diastolic pressure. FMA-UE, Fugl-Meyer Assessment Upper Extremity. MBI, Modified Barthel Index. ^a^ Using the χ^2^ test; ^b^ Using the Mann–Whitney U test.

### 3.2 Model construction

**Figure 2:**
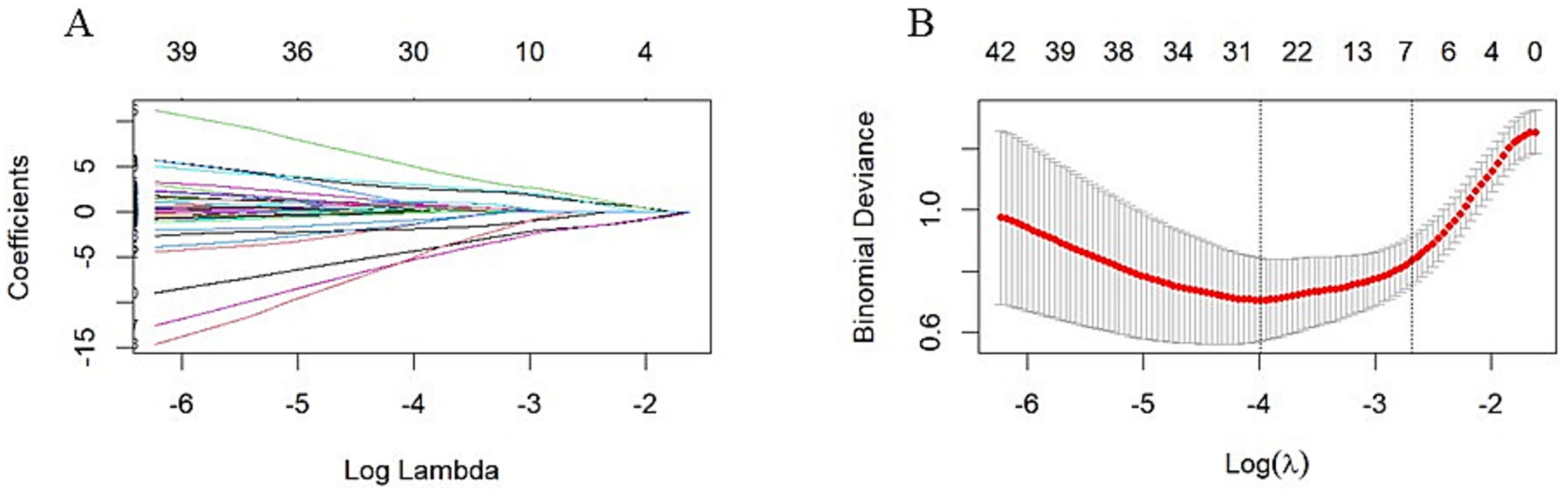
LASSO Regression Analysis with 10-fold Cross-Validation for Predicting Motor Functional Impairment Post-Stroke. **(A)** LASSO Regression Coefficient Pathway Plot: This plot illustrates how the regression coefficients of various features change with the regularization parameter Log Lambda in LASSO regression analysis. Each line represents a feature, with the horizontal axis indicating the Log Lambda value and the vertical axis showing the corresponding regression coefficient. As the Log Lambda value increases, more feature coefficients are shrunk toward zero, achieving feature selection. Ultimately, through LASSO regression analysis, we identified seven variables for assessing motor dysfunction post-stroke, including: A_A_dxy_DLPFC_to_Temporal, A_UA_dxy_Temporal_to_Frontopolar, A_UA_oxy_DLPFC_to_PSMC, A_UA_total_Temporal_to_DLPFC, UA_UA_dxy_Temporal_to_SMA, UA_UA_dxy_Temporal_to_Frontopolar, and UA_UA_total_PSMC_to_PMC. **(B)** Bias-Variance Tradeoff Plot of LASSO Regression: This plot shows the binomial deviance of the model as it changes with Log Lambda in LASSO regression analysis. The red line represents the average deviance, and the gray area indicates the standard error range of the deviance. The horizontal axis is Log Lambda, and the vertical axis is binomial deviance. The two vertical dashed lines in the plot represent the Log Lambda corresponding to the minimum deviance (lambda.min) and the Log Lambda one standard error above the minimum (lambda.1se), respectively. The optimal Log Lambda value is typically chosen near the lowest point of the deviance curve to balance the model’s bias and variance. In this study, we selected seven variables that demonstrated the best predictive performance in cross-validation and were statistically significant (*p* < 0.05).

**Figure 3:**
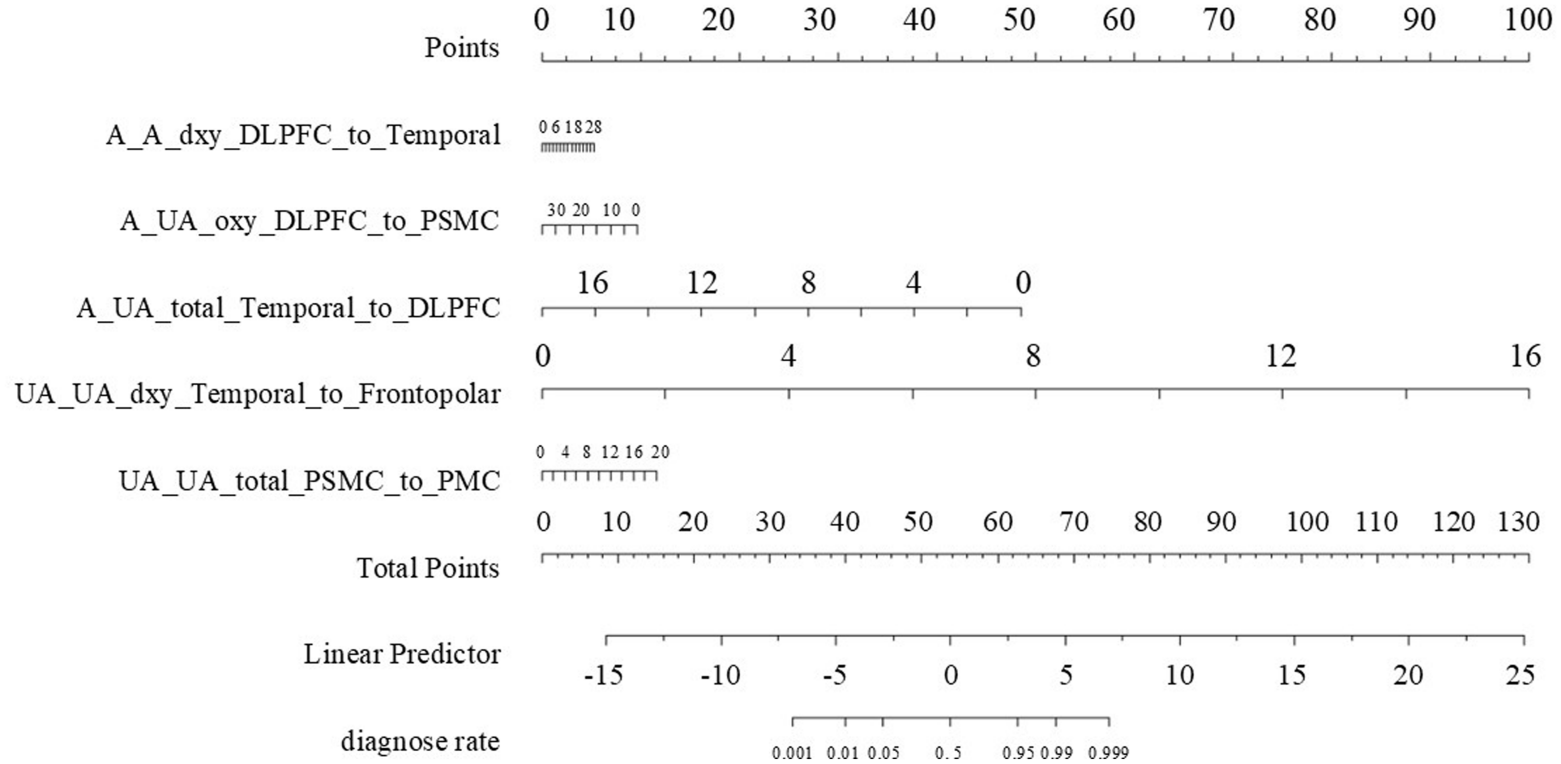
The nomogram. Points Axis: Represents the scores corresponding to each independent risk factor, reflecting its contribution to the prediction probability. Total Points Axis: Accumulates the scores of each risk factor. The higher the total score, the greater the risk of functional impairment. Risk Factor Scales: A_A_dxy_DLPFC_to_Temporal: The numbers of functional connectivity edges from the affected side DLPFC to the temporal lobe, ranging from 0 to 28.A_UA_oxy_DLPFC_to_PSMC: The numbers of functional connectivity edges from the affected side DLPFC to PSMC, ranging from 0 to 30.A_UA_total_Temporal_to_DLPFC: The numbers of functional connectivity edges from the affected side temporal lobe to DLPFC, ranging from 0 to 16.UA_UA_dxy_Temporal_to_Frontopolar: The numbers of functional connectivity edges from the unaffected side temporal lobe to the frontopolar, ranging from 0 to 16.UA_UA_total_PSMC_to_PMC: The numbers of functional connectivity edges from the unaffected side PSMC to PMC, ranging from 0 to 20. Linear Predictor Axis: A linear predictor value calculated based on the total score, used for further calculation of the prediction probability. Diagnose Rate Axis: The probability of no improvement in upper limb motor function 3 months after stroke, as determined by the linear predictor value.

Figures 2A,B illustrate the results of the Lasso regression analysis for feature variable selection. The Lasso regression coefficient plot in Figure 2A shows all potential risk factors, with each risk factor corresponding to a curve, the vertical axis of which represents the regression coefficient of the predictor, and the horizontal axis represents log(λ). Figure 2B displays the bias plot, where the lowest point of the curve corresponds to the optimal lambda parameter. In this study, through Lasso regression analysis, we ultimately identified 7 variables for assessing motor dysfunction post-stroke, including: A_A_dxy_DLPFC_to_Temporal (the numbers of brain functional connectivity edges from affected side DLPFC to affected side Temporal under deoxygenated hemoglobin monitoring level), A_UA_dxy_Temporal_to_Frontopolar (the numbers of brain functional connectivity edges from affected side Temporal to unaffected side Frontopolar under deoxygenated hemoglobin monitoring level), A_UA_oxy_DLPFC_to_PSMC (the numbers of brain functional connectivity edges from the affected side DLPFC to the unaffected side PSMC under oxyhemoglobin monitoring level), A_UA_total_Temporal_to_DLPFC (the numbers of brain functional connectivity edges from affected side Temporal to unaffected side DLPFC under total hemoglobin monitoring level), UA_UA_dxy_Temporal_to_SMA (the numbers of brain functional connectivity edges from unaffected side Temporal to unaffected side SMA under deoxygenated hemoglobin monitoring level), UA_UA_dxy_Temporal_to_Frontopolar (the numbers of brain functional connectivity edges from unaffected side Temporal to unaffected side Frontopolar under deoxygenated hemoglobin monitoring level), and UA_UA_total_PSMC_to_PMC (the numbers of brain functional connectivity edges from unaffected side PSMC to unaffected side PMC under total hemoglobin monitoring level). The selection of these seven variables was based on several criteria: First, these variables demonstrated significant statistical association with motor function recovery post-stroke (*p*-values <0.05), indicating their importance in predicting outcomes. Second, the inclusion of these variables provided the best predictive performance in cross-validation, effectively balancing model bias and variance. Lastly, the optimal number of variables was determined by examining the trade-off between model complexity and predictive accuracy, ensuring that the final model was both robust and interpretable. Subsequently, we used logistic regression to analyze these 7 predictive variables, calculating their weights and *p*-values (*p*-values <0.05). According to the data in Table 3, we found that the *p*-values for A_UA_dxy_Temporal_to_Frontopolar and UA_UA_dxy_Temporal_to_SMA did not reach the level of significance (*p*-values >0.05), thus excluding these two variables. Ultimately, we identified 5 independent risk factors related to the upper limb motor function scores of ischemic stroke: A_A_dxy_DLPFC_to_Temporal, A_UA_oxy_DLPFC_to_PSMC, A_UA_total_Temporal_to_DLPFC, UA_UA_dxy_Temporal_to_Frontopolar, and UA_UA_total_PSMC_to_PMC, all with *p*-values <0.05. As shown in Table 4, based on these risk factors, we developed a nomogram, the details of which can be seen in Figure 3.

**Table 3:** Original model coefficients.

	*B*	SE	OR	CI	*Z*	*P*
A_A_dxy_DLPFC_L_to_Temporal_L	0.54	0.25	1.71	1.10–2.65	2.14	0.033
A_UA_dxy_Temporal_L_to_Frontopolar_R	0.84	0.46	2.31	1.26–4.23	1.83	0.068
A_UA_oxy_DLPFC_L_to_PSMC_R	−0.23	0.10	0.79	0.67–0.93	−2.25	0.024
A_UA_total_Temporal_L_to_DLPFC_R	−0.38	0.17	0.69	0.50–0.95	−2.23	0.026
UA-UA_dxy_Temporal_R_to_SMA_R	−0.10	0.10	0.90	0.74–1.09	−1.02	0.309
UA_UA_dxy_Temporal_R_to_Frontopolar_R	−0.87	0.36	0.42	0.23–0.77	−2.40	0.016
UA_UA_total_PSMC_R_to_PMC_R	0.47	0.21	1.60	1.08–2.38	2.25	0.024

B, Regression Coefficient; SE, Standard Error; OR, Odds Ratio; CI, Confidence Interval.

A_A_dxy_DLPFC_to_Temporal the numbers of brain functional connectivity edges from affected side DLPFC to affected side Temporal under deoxygenated hemoglobin monitoring level.

A_UA_dxy_Temporal_to_Frontopolar the numbers of brain functional connectivity edges from affected side Temporal to unaffected side Frontopolar under deoxygenated hemoglobin monitoring level.

A_UA_oxy_DLPFC_to_PSMC the numbers of brain functional connectivity edges from the affected side DLPFC to the unaffected side PSMC under oxyhemoglobin monitoring level.

A_UA_total_Temporal_to_DLPFC the numbers of brain functional connectivity edges from affected side Temporal to unaffected side DLPFC under total hemoglobin monitoring level.

UA_UA_dxy_Temporal_to_SMA the numbers of brain functional connectivity edges from unaffected side Temporal to unaffected side SMA under deoxygenated hemoglobin monitoring level.

UA_UA_dxy_Temporal_to_Frontopolar the numbers of brain functional connectivity edges from unaffected side Temporal to unaffected side Frontopolar under deoxygenated hemoglobin monitoring level.

UA_UA_total_PSMC_to_PMC the numbers of brain functional connectivity edges from unaffected side PSMC to unaffected side PMC under total hemoglobin monitoring level.

**Table 4:** Final model coefficients.

	*B*	SE	OR	CI	*Z*	*P*
A_A_dxy_DLPFC_L_to_Temporal_L	0.74	0.24	2.09	1.26–3.46	3.07	0.002
A_UA_oxy_DLPFC_L_to_PSMC_R	−0.26	0.09	0.77	0.65–0.90	−2.88	0.004
A_UA_total_Temporal_L_to_DLPFC_R	−0.34	0.13	0.71	0.54–0.93	−2.59	0.009
UA_UA_dxy_Temporal_R_to_Frontopolar_R	−0.63	0.24	0.53	0.34–0.82	−2.65	0.008
UA_UA_total_PSMC_R_to_PMC_R	0.49	0.19	1.63	1.06–2.51	2.62	0.009

B, Regression Coefficient; SE, Standard Error; OR, Odds Ratio; CI, Confidence Interval.

A_A_dxy_DLPFC_to_Temporal the numbers of brain functional connectivity edges from affected side DLPFC to affected side Temporal under deoxygenated hemoglobin monitoring level.

A_UA_dxy_Temporal_to_Frontopolar the numbers of brain functional connectivity edges from affected side Temporal to unaffected side Frontopolar under deoxygenated hemoglobin monitoring level.

A_UA_oxy_DLPFC_to_PSMC the numbers of brain functional connectivity edges from the affected side DLPFC to the unaffected side PSMC under oxyhemoglobin monitoring level.

A_UA_total_Temporal_to_DLPFC the numbers of brain functional connectivity edges from affected side Temporal to unaffected side DLPFC under total hemoglobin monitoring level.

UA_UA_dxy_Temporal_to_SMA the numbers of brain functional connectivity edges from unaffected side Temporal to unaffected side SMA under deoxygenated hemoglobin monitoring level.

UA_UA_dxy_Temporal_to_Frontopolar the numbers of brain functional connectivity edges from unaffected side Temporal to unaffected side Frontopolar under deoxygenated hemoglobin monitoring level.

UA_UA_total_PSMC_to_PMC the numbers of brain functional connectivity edges from unaffected side PSMC to unaffected side PMC under total hemoglobin monitoring level.

#### 3.2.1 Overall introduction to the nomogram

The nomogram we constructed is based on independent predictive factors identified through logistic regression analysis, designed to predict the probability of upper limb motor dysfunction scores in ischemic stroke patients. It integrates multiple variables related to the recovery of upper limb motor function into an intuitive graphical tool, providing clinicians with a convenient method to assess patient risk and formulate personalized rehabilitation plans.

#### 3.2.2 Specific significance and understanding of each sub-figure

##### 3.2.2.1 Points

A_A_dxy_DLPFC_to_Temporal: Represents the score scale of the number of brain functional connectivity edges from the affected side DLPFC to the affected side Temporal under deoxygenated hemoglobin monitoring conditions, ranging from 6 to 28. For example, if a patient’s value for this variable is 28, they score 6 points.

A_UA_oxy_DLPFC_to_PSMC: Represents the score scale of the number of brain functional connectivity edges from the affected side DLPFC to the unaffected side PSMC under oxyhemoglobin monitoring conditions, ranging from 0 to 30. For example, if a patient’s value for this variable is 0, they score 10 points.

A_UA_total_Temporal_to_DLPFC: Represents the score scale of the number of brain functional connectivity edges from the affected side Temporal to the unaffected side DLPFC under total hemoglobin monitoring conditions, ranging from 0 to 18. For example, if a patient’s value for this variable is 0, they score 50 points.

UA_UA_dxy_Temporal_to_Frontopolar: Represents the score scale of the number of brain functional connectivity edges from the unaffected side Temporal to the unaffected side Frontopolar under deoxygenated hemoglobin monitoring conditions, ranging from 0 to 16. For example, if a patient’s value for this variable is 2, they score 10 points.

UA_UA_total_PSMC_to_PMC: Represents the score scale of the number of brain functional connectivity edges from the unaffected side PSMC to the unaffected side PMC under total hemoglobin monitoring conditions, ranging from 0 to 20. For example, if a patient’s value for this variable is 4, they score 2 points.

##### 3.2.2.2 Total points

This is the total score scale obtained by adding up the scores of the above variables, ranging from 0 to 130. After summing the scores of each variable, the patient’s total score is located on this scale. The higher the total score, the higher the risk probability of no improvement in upper limb motor function 3 months after the onset. For example, the total score for the aforementioned patient is 6 + 10 + 50 + 10 + 2 = 78 points.

##### 3.2.2.3 Linear predictor

This scale ranges from −15 to 25 and is an intermediate variable calculated based on the logistic regression model. The values of the variables for each patient are transformed into a linear predictor through the logistic regression model, which is further associated with the predicted risk probability. Clinicians can sum the scores of each variable in the nomogram to find the corresponding linear predictor value, thereby understanding how the model integrates multiple variables’ information into a risk prediction. For example, the aforementioned patient with a total score of 78 points corresponds to a Linear Predictor of 8.

##### 3.2.2.4 Diagnose rate

This is the final predicted diagnosis rate scale, ranging from 0.001 to 0.999. It represents the predicted probability of no improvement in upper limb motor function 3 months after the onset. For instance, if the aforementioned patient’s Linear Predictor is 8, the corresponding value on the diagnosis rate scale is 0.999, indicating that the predicted probability of no improvement in upper limb motor function 3 months after the onset is 99.9%. Clinicians can use this diagnosis rate, in conjunction with the patient’s specific situation, to develop more targeted rehabilitation treatment plans and follow-up strategies.

### 3.3 Model validation

**Figure 4:**
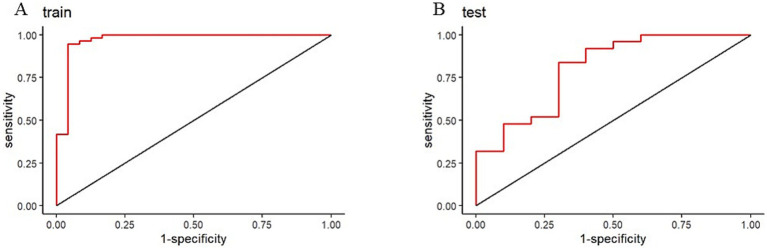
Receiver operating characteristic (ROC) curves for nomogram. **(A)** Training ROC. **(B)** Validation ROC.

**Figure 5:**
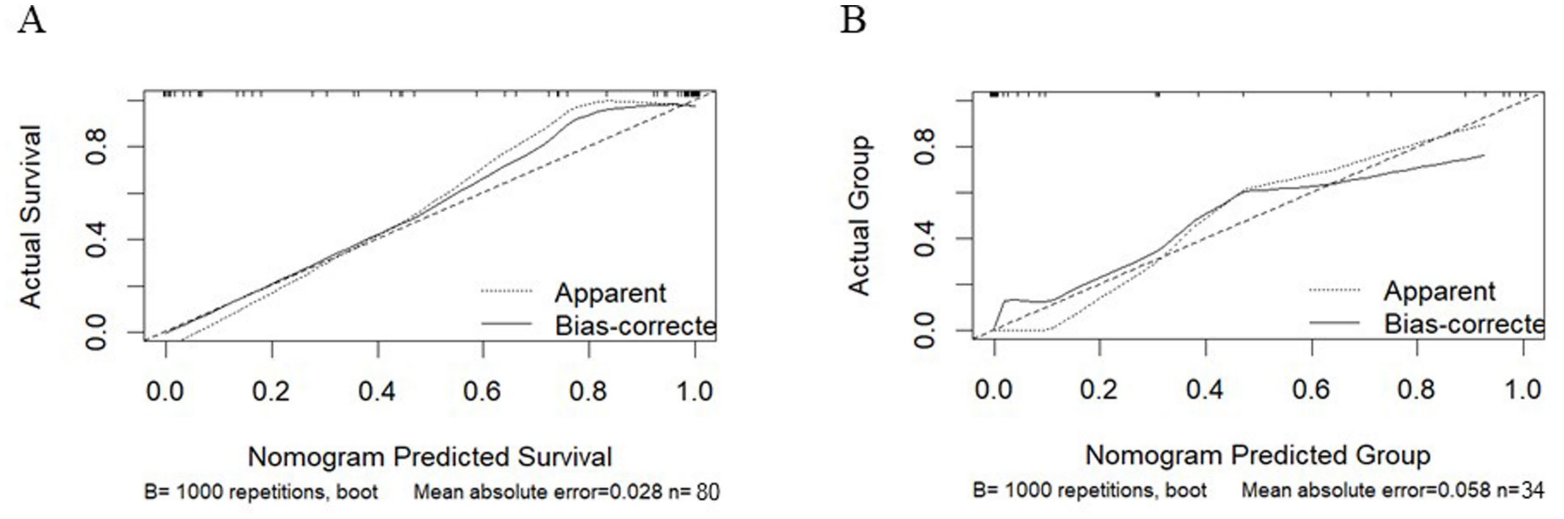
Calibration curves for the training cohort and the validation cohort. **(A)** Training Cohort. **(B)** Validation Cohort.

**Figure 6:**
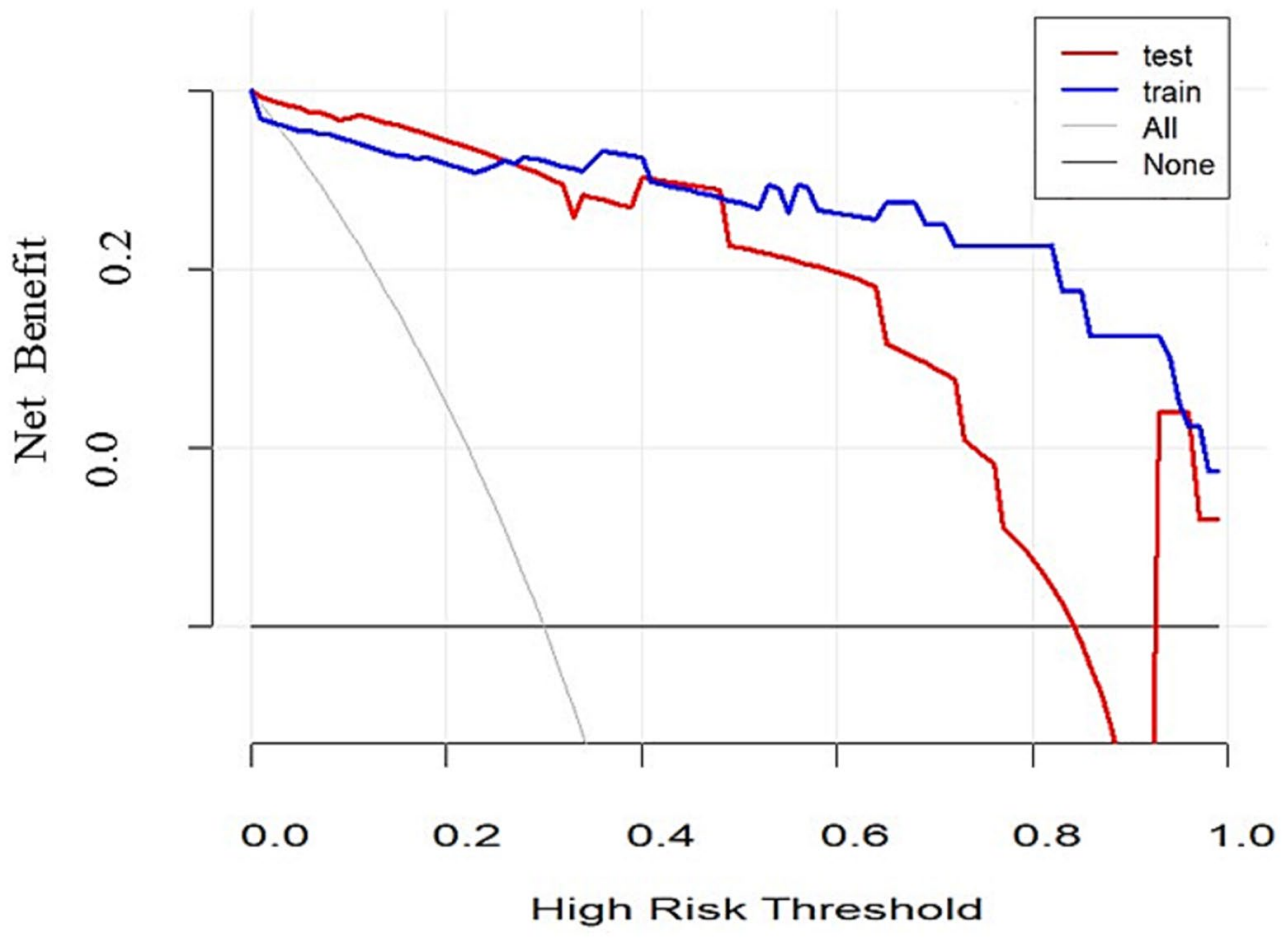
Decision-curve analysis.

To validate the discriminative power of our model, we calculated the area under the ROC curve (AUC). In Figure 4A, the AUC for the training dataset reached 0.971 (95% confidence interval 0.913 to 1), and in Figure 4B, the AUC for the testing dataset was 0.804 (95% confidence interval 0.531 to 0.956). Figure 5 shows that the model has good predictive ability in both the training and validation sets, and after bias correction, the model’s predictive performance remains stable across different thresholds. Figure 6 presents the decision curve analysis (DCA) results of the model, which shows that the model achieved positive clinical net benefit in both the validation and development cohorts.

## 4 Discussion

This study successfully identified independent risk factors associated with upper limb motor functional recovery in ischemic stroke patients 3 months post-stroke using fNIRS technology and constructed a predictive model. The results indicate that the five independent risk factors selected through LASSO regression—A_A_dxy_DLPFC_to_Temporal, A_UA_oxy_DLPFC_to_PSMC, A_UA_total_Temporal_to_DLPFC, UA_UA_dxy_Temporal_to_Frontopolar, and UA_UA_total_PSMC_to_PMC—can effectively predict the probability of upper limb motor dysfunction 3 months after stroke. This finding provides clinicians with an assessment tool based on objective physiological data, aiding in the more accurate prediction of patients’ rehabilitation potential and the formulation of personalized rehabilitation plans (16, 17).

Compared to previous research, our model boasts distinct advantages. Some existing models mainly rely on clinical features like age and NIHSS scores for prediction (18). In contrast, our fNIRS – based model can offer more direct physiological data on brain function. Models relying solely on clinical data cannot fully mirror the real – time changes in brain neural activities during recovery.

Regarding imaging techniques, while structural and functional MRI can evaluate brain integrity and functional reorganization (19, 20), fNIRS is more portable, less expensive, and easier to operate in clinical settings. Moreover, compared with some machine – learning – based predictive models that demand a large amount of complex data (18), ours simplifies input data yet still maintains excellent predictive performance.

Our model demonstrated good discriminative ability in both the training and validation cohorts, indicating high stability and generalizability. The AUC value of the ROC curve approaching 1 suggests high predictive accuracy. Moreover, the decision curve analysis (DCA) results showed that the model had good clinical net benefit across different thresholds, further validating its clinical application value.

In terms of functional recovery after stroke, several predictive models have been proposed. These models aim to predict the likelihood of recovery by analyzing patients’ clinical characteristics, neuroimaging data, and other biomarkers. For instance, a prospective cohort study showed that age and the National Institutes of Health Stroke Scale (NIHSS) could be used to develop a model predicting gait and upper limb functional recoveries (21). Additionally, systematic reviews and meta-analyses have demonstrated that existing prognostic models perform well in predicting full recovery in patients with ischemic stroke (22).

Regarding functional connectivity, studies have indicated that functional reorganization of brain regions is closely related to motor recovery after stroke (23). Research using functional near-infrared spectroscopy (fNIRS) has found that significant changes in resting-state functional connectivity patterns of the motor cortex occur during the recovery process, potentially associated with improvements in upper limb function (24). Structural and functional magnetic resonance imaging (MRI) has been used to assess the integrity and functional reorganization of the brain, showing good predictive effects on hand motor outcomes (25). Studies have also found a significant correlation between white matter integrity and cortical functional connectivity with motor recovery (26). Furthermore, functional connectivity analysis based on electroencephalography (EEG) has been proposed as a biomarker for predicting motor recovery, demonstrating the relationship between reorganization of brain networks and functional improvement during rehabilitation (27). Machine learning methods have also been applied to predict functional recovery in stroke patients, with studies indicating that these models have high accuracy in predicting clinically significant motor functional improvement (28).

Despite the positive results of this study, there are some limitations. Firstly, the sample size is relatively small, which may affect the model’s universality. Secondly, this study is a single-center study, and further validation of the model’s predictive ability is needed in multicenter studies with larger sample sizes. Additionally, this study only considered fNIRS data; future studies could consider combining other biomarkers or clinical data to improve the model’s predictive accuracy.

It is worth noting that future research should fully consider the impact of the vascular distribution of acute ischemic stroke on functional recovery. Clinical reports have shown that cerebral infarcts in the territory of the posterior cerebral artery have a better prognosis than those in the territory of the middle cerebral artery (29). However, in this study, we did not stratify the enrolled patients based on vascular distribution. In light of this, we plan to increase the sample size in our future work and conduct subgroup analyses based on vascular distribution to more accurately assess its impact on functional recovery.

In addition, future research should focus on evaluating upper limb dysfunction in lacunar versus non-lacunar ischemic strokes. Lacunar infarcts, which have the best functional prognosis among stroke subtypes, are particularly notable even in pure motor stroke, the lacunar syndrome with the most severe functional impairment (30). These factors undoubtedly have a significant impact on functional recovery after stroke and are worthy of in-depth exploration in future studies.

In conclusion, this study offers a predictive model for upper limb motor dysfunction following ischemic stroke based on fNIRS data, providing a new tool for clinical assessment and treatment. Future research should further explore and refine this model to achieve more precise stroke rehabilitation evaluations.

## 5 Conclusion

By utilizing functional near-infrared spectroscopy (fNIRS) data and employing LASSO regression and logistic regression analysis, this study constructed an effective predictive model for upper limb motor dysfunctions after ischemic stroke and developed a nomogram prediction tool. This tool demonstrated good discriminative ability and clinical net benefit in both the training and validation cohorts. The findings highlight the potential of fNIRS in stroke rehabilitation assessment and provide clinicians with an assessment tool based on objective physiological data, facilitating the development of personalized rehabilitation plans. However, due to the limitations of sample size and single-center study, the conclusions of this study require further validation in a broader patient population and multicenter studies.

## Data Availability

The original contributions presented in the study are included in the article/supplementary material, further inquiries can be directed to the corresponding author.

## References

[1] Sun J Sun R Li C Luo X Chen J Hong J. NgR1 pathway expression in cerebral ischemic Sprague-Dawley rats with cognitive impairment. Iran J Basic Med Sci. (2021) 24:767–75. doi: 10.22038/ijbms.2021.53316.12011, PMID: 34630954 PMC8487595

[2] Puhr-Westerheide D Froelich MF Solyanik O Gresser E Reidler P Fabritius MP. Cost-effectiveness of short-protocol emergency brain MRI after negative non-contrast CT for minor stroke detection. Eur Radiol. (2022) 32:1117–26. doi: 10.1007/s00330-021-08222-z, PMID: 34455484 PMC8794930

[3] Hu JX Ma WJ He LY Zhang CH Zhang C Wang Y. Macrophage migration inhibitory factor (MIF) acetylation protects neurons from ischemic injury. Cell Death Dis. (2022) 13:466. doi: 10.1038/s41419-022-04918-2, PMID: 35585040 PMC9117661

[4] Li Q Hu S Mo Y Chen H Meng C Zhan L. Regional homogeneity alterations in multifrequency bands in patients with basal ganglia stroke: a resting-state functional magnetic resonance imaging study. Front Aging Neurosci. (2022) 14:938646. doi: 10.3389/fnagi.2022.938646 36034147 PMC9403766

[5] Zhou Y Zhang S Fan X. Role of polyphenols as antioxidant supplementation in ischemic stroke. Oxidative Med Cell Longev. (2021) 2021:5471347. doi: 10.1155/2021/5471347 PMC8253632 34257802

[6] Luo Z Zhou Y He H Lin S Zhu R Liu Z. Synergistic effect of combined Mirror therapy on upper extremity in patients with stroke: a systematic review and Meta-analysis. Front Neurol. (2020) 11:155. doi: 10.3389/fneur.2020.00155 32300326 PMC7144801

[7] Ortega-Martinez A Von Lühmann A Farzam P Rogers D Mugler EM Boas DA. Multivariate Kalman filter regression of confounding physiological signals for real-time classification of fNIRS data. Neurophotonics. (2022) 9:025003. doi: 10.1117/1.NPh.9.2.025003, PMID: 35692628 PMC9174890

[8] Veliev F Han Z Kalita D Briançon-Marjollet A Bouchiat V Delacour C. Recording spikes activity in cultured hippocampal neurons using flexible or transparent graphene transistors. Front Neurosci. (2017) 11:466. doi: 10.3389/fnins.2017.00466 28894412 PMC5581354

[9] Gao Y Chao H Cavuoto L Yan P Kruger U Norfleet JE. Deep learning-based motion artifact removal in functional near-infrared spectroscopy. Neurophotonics. (2022) 9:041406. doi: 10.1117/1.NPh.9.4.041406, PMID: 35475257 PMC9034734

[10] Xie W Ma X Xu G Wang Y Huang W Liu M. Development and validation of a nomogram for the risk prediction of malignant cerebral edema after acute large hemispheric infarction involving the anterior circulation. Front Neurol. (2023) 14:1221879. doi: 10.3389/fneur.2023.1221879, PMID: 37780698 PMC10538642

[11] Lee JJ Shin JH. Predicting clinically significant improvement after robot-assisted upper limb rehabilitation in subacute and chronic stroke. Front Neurol. (2021) 12:668923. doi: 10.3389/fneur.2021.668923 34276535 PMC8281036

[12] Valladares B Kundert RG Pohl J Held JPO Luft AR Veerbeek JM. The association between dexterity and upper limb impairment during stroke recovery. Front Neurol. (2024) 15:1429929. doi: 10.3389/fneur.2024.1429929 39224885 PMC11367986

[13] Arya KN Verma R Garg RK. Estimating the minimal clinically important difference of an upper extremity recovery measure in subacute stroke patients. Top Stroke Rehabil. (2011) 18:599–610. doi: 10.1310/tsr18s01-599, PMID: 22120029

[14] Wang X Luo Z Zhang M Zhao W Xie S Wong SF. The interaction between changes of muscle activation and cortical network dynamics during isometric elbow contraction: a sEMG and fNIRS study. Front Bioeng Biotechnol. (2023) 11:1176054. doi: 10.3389/fbioe.2023.1176054 37180038 PMC10167054

[15] Li L Zhang M Chen Y Wang K Zhou G Huang Q. TAGL: temporal-guided adaptive graph learning network for coordinated movement classification. IEEE Trans Indus Inform. (2024) 20:12554–64. doi: 10.1109/TII.2024.3423311

[16] Honma K Honda Y Nagase M Nakao Y Harada T Sasanuma N. Impact of skeletal muscle mass on functional prognosis in acute stroke: a cohort study. J Clin Neurosci. (2023) 112:43–7. doi: 10.1016/j.jocn.2023.04.006 37062242

[17] Werner RA. Predicting outcome after acute stroke with the functional Independence measure. Top Stroke Rehabil. (1994) 1:30–9. PMID: 27680953 10.1080/10749357.1994.11754032

[18] Hassan A Gulzar Ahmad S Ullah Munir E Ali Khan I Ramzan N. Predictive modelling and identification of key risk factors for stroke using machine learning. Sci Rep. (2024) 14:11498. doi: 10.1038/s41598-024-61665-4, PMID: 38769427 PMC11106277

[19] Johansen-Berg H Rushworth MF. Using diffusion imaging to study human connectional anatomy. Annu Rev Neurosci. (2009) 32:75–94. doi: 10.1146/annurev.neuro.051508.135735, PMID: 19400718

[20] Rieke JD Matarasso AK Yusufali MM Ravindran A Alcantara J White KD. Development of a combined, sequential real-time fMRI and fNIRS neurofeedback system to enhance motor learning after stroke. J Neurosci Methods. (2020) 341:108719. doi: 10.1016/j.jneumeth.2020.108719, PMID: 32439425

[21] Kwah LK Harvey LA Diong J Herbert RD. Models containing age and NIHSS predict recovery of ambulation and upper limb function six months after stroke: an observational study. J Physiother. (2013) 59:189–97. doi: 10.1016/S1836-9553(13)70183-8, PMID: 23896334

[22] Jampathong N Laopaiboon M Rattanakanokchai S Pattanittum P. Prognostic models for complete recovery in ischemic stroke: a systematic review and meta-analysis. BMC Neurol. (2018) 18:26. doi: 10.1186/s12883-018-1032-5, PMID: 29523104 PMC5845155

[23] Olafson ER Jamison KW Sweeney EM Liu H Wang D Bruss JE. Functional connectome reorganization relates to post-stroke motor recovery and structural and functional disconnection. Neuroimage. (2021) 245:118642. doi: 10.1016/j.neuroimage.2021.118642 34637901 PMC8805675

[24] Arun KM Smitha KA Sylaja PN Kesavadas C. Identifying resting-state functional connectivity changes in the motor cortex using fNIRS during recovery from stroke. Brain Topogr. (2020) 33:710–9. doi: 10.1007/s10548-020-00785-2, PMID: 32685998

[25] Horn U Grothe M Lotze M. MRI biomarkers for hand-motor outcome prediction and therapy monitoring following stroke. Neural Plast. (2016) 2016:9265621. doi: 10.1155/2016/9265621 PMID: 27747108 PMC5056270

[26] Lee J Kim H Kim J Chang WH Kim YH. Multimodal imaging biomarker-based model using stratification strategies for predicting upper extremity motor recovery in severe stroke patients. Neurorehabil Neural Repair. (2022) 36:217–26. doi: 10.1177/15459683211070278 34970925

[27] Philips GR Daly JJ Príncipe JC. Topographical measures of functional connectivity as biomarkers for post-stroke motor recovery. J Neuroeng Rehabil. (2017) 14:67. doi: 10.1186/s12984-017-0277-3, PMID: 28683745 PMC5501348

[28] Thakkar HK Liao WW Wu CY Hsieh YW Lee TH. Predicting clinically significant motor function improvement after contemporary task-oriented interventions using machine learning approaches. J Neuroeng Rehabil. (2020) 17:131. doi: 10.1186/s12984-020-00758-3, PMID: 32993692 PMC7523081

[29] Arboix A Arbe G García-Eroles L Oliveres M Parra O Massons J. Infarctions in the vascular territory of the posterior cerebral artery: clinical features in 232 patients. BMC Res Notes. (2011) 4:329. doi: 10.1186/1756-0500-4-329 21899750 PMC3180463

[30] Arboix A Massons J García-Eroles L Targa C Comes E Parra O. Clinical predictors of lacunar syndrome not due to lacunar infarction. BMC Neurol. (2010) 10:31. doi: 10.1186/1471-2377-10-31 20482763 PMC2877662

